# Neutrophil Extracellular Traps (NETs): Opportunities for Targeted Therapy

**DOI:** 10.32607/actanaturae.11503

**Published:** 2021

**Authors:** D. V. Volkov, G. V. Tetz, Y. P. Rubtsov, A. V. Stepanov, A. G. Gabibov

**Affiliations:** Shemyakin-Ovchinnikov Institute of Bioorganic Chemistry RAS, Moscow, 117997 Russia; Pavlov First State Medical University of St. Petersburg, St Petersburg, 197022 Russia

**Keywords:** cancer, tumor microenvironment, neutrophils, NETosis

## Abstract

Antitumor therapy, including adoptive immunotherapy, inevitably faces powerful counteraction from advanced cancer. If hematological malignancies are currently amenable to therapy with CAR-T lymphocytes (T-cells modified by the chimeric antigen receptor), solid tumors, unfortunately, show a significantly higher degree of resistance to this type of therapy. As recent studies show, the leading role in the escape of solid tumors from the cytotoxic activity of immune cells belongs to the tumor microenvironment (TME). TME consists of several types of cells, including neutrophils, the most numerous cells of the immune system. Recent studies show that the development of the tumor and its ability to metastasize directly affect the extracellular traps of neutrophils (neutrophil extracellular traps, NETs) formed as a result of the response to tumor stimuli. In addition, the nuclear DNA of neutrophils – the main component of NETs – erects a spatial barrier to the interaction of CAR-T with tumor cells. Previous studies have demonstrated the promising potential of deoxyribonuclease I (DNase I) in the destruction of NETs. In this regard, the use of eukaryotic deoxyribonuclease I (DNase I) is promising in the effort to increase the efficiency of CAR-T by reducing the NETs influence in TME. We will examine the role of NETs in TME and the various approaches in the effort to reduce the effect of NETs on a tumor.

## INTRODUCTION

Unlike hematologic cancers, malignant solid tumors form a closed structure consisting of several layers. Cancer cells residing in the tumor center and carrying adhesion receptors on their surface are linked by tunneling nanotubes and communicate with each other through autocrine and paracrine signals transmitted via soluble factors and the extracellular matrix. A layer forming another niche (involving vessels, cancer-associated fibroblasts and stromal cells receiving signals via adhesion receptors and soluble factors) lies closer to the periphery. Farther away from the tumor’s center lies a confined layer that is reached by stimulation or inhibition signals from tumor cells and includes the neovasculature, intratumoral lymph nodes, immune cells, cancer-associated fibroblasts, the extracellular matrix, and nerve endings. The proximal (with respect to the normal tissue) layer that involves the nearest lymphatic and blood vessels, immune cells, and proximal lymphoid elements is considered to be the outermost layer. The additional levels of tumor cell architecture that influence cancer development refer to metastatic foci. The so-called confined layer is considered a boundary of the tumor microenvironment. The neoplasm’s complex structural morphology requires the engineering of targeted therapy based on a significant mechanistic understanding of therapeutic agents’ penetration directly to transforming cells [1, 2, 3, 4, 5].

The major portion of TME consists of the host’s immune cells, with neutrophils being the most numerous group. Inflammation develops within the tumor growth region, and the signals released by malignant and tumor-associated cells recruit neutrophils, which are converted to tumor-associated neutrophils (TANs). They belong to the group of myeloid-derived suppressor cells (MDSCs). MDSCs can also manifest in noncancer cases; however, these cells inhibit the protective antitumor immune response in cancer patients. TANs also receive cell death (cellular suicide) signals, which induces a specific type of cell death accompanied by the release of a large quantity of genomic DNA, as well as the proteins and enzymes associated with it, which eventually form NETs. The composition of NETs varies depending on the type of the initial stimulus/a combination of stimuli. The chromosomal DNA network is an invariable part of NETs. This has led researchers to suggest that deoxyribonucleases can be used to efficiently degrade NETs. Indeed, recent studies have demonstrated that DNase I administered to experimental mice slows the progression of a primary tumor, inhibits the metastatic potential of tumor cells, and increases animals’ lifespan. The hopeful results of research focusing on the administration of purified DNase I to mice have driven the elaboration of novel methods for the delivery of DNase I into the body.

## FORMATION OF NETS AND THEIR COMPOSITION

Neutrophil extracellular traps were discovered as one of the defense mechanisms of neutrophils in response to bacterial infection [6]. Released NETs impede the transmission of pathogens in the blood flow and kill pathogenic microorganisms [6, 7]. Later, NETs were also found in tumor biopsy specimens from patients with different types of cancer. Their presence correlated with poor prognosis in patients [8, 9, 10, 11]. This discovery has spurred active research into the role played by NETs in oncogenesis.

**Fig. 1:**
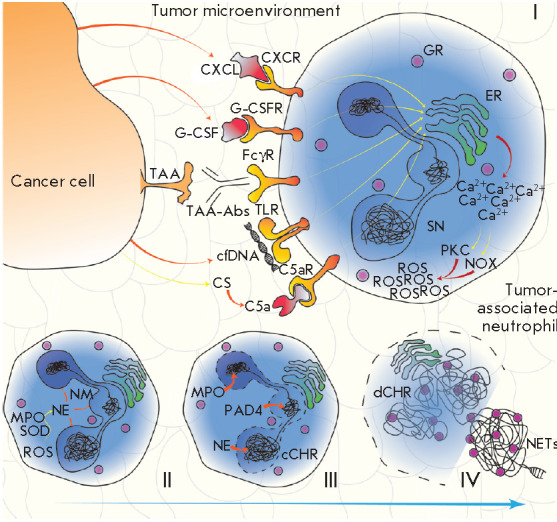
The signaling pathway of NOX-dependent NETosis. Various cancer-associated stimuli increase the cytoplasmic Ca^2+^ concentration in TANs, which results in the activation of PKC and NOX and, therefore, leads to intracellular production of ROS (I). As SOD and MPO interact, ROS are converted into HClO, leading to the activation of NE (II). NE promotes NM degradation, and then PAD4, MPO, and NE ensure chromatin decondensation and its mixing with cytoplasmic granules (III); the resulting mixture (in the form of NETs) is released into the extracellular space during NETosis (IV). Abbreviations: TAA – tumor-associated antigen; cfDNA – cell-free DNA; TAA-Abs – anti-TAA antibodies; FcγR – receptor for the fragment crystallizable region of IgG; TLR – toll-like receptor; CXCL – cytokine belonging to the CXC family; CXCR – CXCL receptor; ER – endoplasmic reticulum; GR – granule; G-CSF – granulocyte colony-stimulating factor; G-CSFR – G-CSF receptor; CS – complement system; C5a – complement component 5a; C5aR – C5a receptor; SN – segmented nucleus; NM – nuclear membrane; NE – neutrophil elastase; MPO – myeloperoxidase; SOD – superoxide dismutase; ROS – reactive oxygen species; PKC – protein kinase C; NOX – NADPH oxidase; cCHR – condensed chromatin; PAD4 – protein arginine deiminase 4; dCHR – decondensed chromatin; NETs – extracellular neutrophil traps

In the best-studied pathway leading to the expulsion of NETs (*Fig. 1*), signal transduction by extracellular signal-regulated kinase (EPK) results in the activation of NADPH oxidase (NOX) (*Fig. 1, I*) and production of superoxide radicals, which are converted to hydrogen peroxide by superoxide dismutase (*Fig. 1, II*) [12]. Myeloperoxidase (MPO) converts hydrogen peroxide to hypochlorous acid, and activates neutrophil elastase (NE) (*Fig. 1, II*). Neutrophil elastase is responsible for the disassembly of the cytoskeleton and nuclear membrane; it allows the nuclear content to mix with the cytoplasm (*Fig. 1, II*) [13]. The conversion of the arginine residues within histones to citrulline (citrullination) by activated protein arginine deiminase (PAD) and proteolytic cleavage of MPO and NE cause chromatin decondensation (*Fig. 1, III*) [14]. Chromatin fibers bind to granules and cytoplasmic proteins, to be eventually expelled from the cell (*Fig. 1, IV*).

Production of reactive oxygen species (ROS) is the key event in NETosis (*Fig. 1, I*). The mitochondrial respiratory chain and NOX contribute independently to the formation of oxygen species. Many different receptors trigger the formation of NETs by activating NOX in the classical suicidal NETosis [15] (*Fig. 1, I*). Identically, phorbol 12-myristate 13-acetate (PMA) mimics diacylglycerol and activates protein kinase C (PKC) [16] and ERK signal transduction, which is similar to the induction of NETs by pathogenic bacteria and fungi. Interestingly, the pathways of PMA-mediated induction of NETosis in cultivated neutrophils can differ significantly [17].

The NOX-independent NETosis pathway is based on the production of mitochondrial ROS promoted by alkaline pH, which increases the inflow of Ca^2+^ [18]. In turn, Ca^2+^ activates SK3, one of the types of small conductance calcium-activated potassium channels (SK), a crucial step in NOX-independent NETosis [19]. PAD4 activation and histone citrullination are clearly visible in NOX-independent NETosis. Calcium ionophores such as ionomycin and A23187 (calcimycin) activate PKC-ζ and, then, PAD4 [16], thus triggering NOX-independent NETosis. Under certain conditions, nuclear and mitochondrial DNA is released via the NOX-independent pathway from live neutrophils. It was shown that ribonucleoprotein immune complexes act upon normal neutrophils or low-density immunosuppressive neutrophils, thus inducing the production of mitochondrial ROS and release of NETs containing mitochondrial DNA from living cells [20]. In patients with sepsis, activated platelets adhere to neutrophils and cause the extrusion of NETs from living cells [21].

Although production of ROS and enzyme activities play different roles in NETosis induction, the different activation pathways result in the formation of NETs exhibiting similar bactericidal capabilities [22].

Along with ionophores and PMA, there are more than a dozen substances capable of inducing NETosis, which can be used *in vitro *to analyze this process [10]. A proteomic analysis of NETs induced by various stimuli has revealed 330 proteins within these NETs; 74 of these proteins were present regardless of the method used for NETosis induction, comprising a pool of key elements that characterizes any type of the known NETs [23, 24].

## THE ROLE OF NETS IN TUMOR PROCESSES

The data on the link between NETs and cancer progression have driven intense research into the functions of NETs in different tumor types. It was reported soon after that NETs have a direct impact on the proliferation of tumor cells through proteases or activating signaling [25, 26, 27, 28].

**Cancer cells are one of the reasons for NETosis**

Cancer cells were shown to be able to induce NETosis both *in vivo *and *in vitro *[11], and the link between TANs and NET formation was also demonstrated [11, 29, 30, 31]. Thus, it has been found *in vitro *that the human pancreatic tumor cell line (AsPC-1) induces NET formation [32]; the extracellular proteins expressed in this cell line are considered to play a crucial role in NETosis. The study has also demonstrated that NETs enhance the endogenous thrombin potential of normal plasma and induce the migration, invasion, and angiogenesis of cancer cells [32]. As shown in another *in vitro *study, extracellular RNAs from Lewis lung carcinoma cells cause NET formation [33].

Neutrophils in mice with chronic myeloid leukemia, breast or lung cancer are more susceptible to NETosis than those in healthy animals. The high susceptibility of neutrophils to NET formation in these pathologies correlates with the systemic effect tumors have on the organism [34, 35].

Neutrophil recruitment by a conditioned medium from hypoxic cancer cells was observed *in vitro*. Cell migration was mediated by high levels of chemokines and HMGB1, which can also generate NETs in the TME [31]. Tohme *et al. *[31] have recently shown that NETs promote tumor cell growth by enhancing their mitochondrial function. Furthermore, tumors implanted subcutaneously grew faster in control mice than in PAD4 knockout (PAD4-KO) ones in these researchers’ experiments. PAD4-deficient mice had fewer hepatic metastases compared to the control group. Recombinant DNase I injected intraperitoneally also reduced the number of metastases in PAD4 wild-type mice. Immunofluorescence staining of tumor tissue slices in PAD4-KO mice showed a very low level of neutrophil infiltration compared to the control. Overall, these data emphasize the pivotal role played by neutrophil recruitment and NET formation in tumor growth and progression [31]. Park *et al. *also revealed a close relationship between metastatic cancer cells, neutrophil recruitment, and NET formation [11]. They showed that metastatic breast cancer cells induce NETosis that maintains metastases due to NETs. Cytokine CXCL1 mediated neutrophil recruitment in tumor in mice with orthotypically transplanted breast cancer cells: 4T1 (metastatic) and 4T07 (non-metastatic). Primary 4T1 tumors were found to contain more neutrophils than 4T07 tumors do. The lower CXCL1 level in 4T1 cells reduced neutrophil infiltration in the tumor. It was shown by immunofluorescence staining of lung tissue slices that NETs form immediately after 4T1 has been injected into the tail vein. Furthermore, metastatic cells released a granulocyte colony-stimulating factor (G-CSF), which induced NETosis around these cells, while antibodies blocking G-CSF significantly reduced NET formation after injection of 4T1 cells [11].

**NETs are involved in circulatory disturbance**

Changes in blood vessels and increased neutrophil infiltration in the heart and kidney resembling the systemic lesions in cancer patients were revealed in RIP1-Tag2 (spontaneous insulinoma) and MMTV-PyMT (breast cancer) transgenic mice. Furthermore, platelet–neutrophil complexes were detected in the kidney of these animals, an indication of NET formation. It is noteworthy that this phenomenon was observed in none of the analyzed healthy mice [36]. It was shown earlier that platelets drive neutrophils to release NETs, thus promoting bacterial death [21]. Olsson *et al*. found that accumulation of NETs in the vasculature was related to the activation of the proinflammatory adhesion molecules ICAM-1, VCAM-1. and E-selectin, as well as the proinflammatory cytokines IL-1b, IL-6 and chemokine CXCL1. DNase I injected to ensure NET degradation normalized renal and cardiac perfusion and prevented vascular occlusion in these organs. The results of this study strongly suggest that NETs mediate the detrimental harmful effects of tumors on distal organs by disrupting tumor vasculature and increasing the likelihood of inflammation in them [36].

In case of pancreatic adenocarcinoma (PA), NETs and platelets play a crucial role in blood hypercoagulation, which increases the risk of venous thromboembolism and cancer-associated thrombosis both in the orthotopic PA model in C57BL/6 mice and in patients [37]. Berger-Achituv *et al*. [8] showed that TANs are found in diagnostic biopsy specimens from children with Ewing sarcoma. In two specimens, NETs were produced due to TANs. These patients had metastases and early tumor recurrence after high-dose chemotherapy, thus indicating that NETs might play a role in the progression of Ewing sarcoma [8]. The association of NETs with altered coagulation in patients with tumors attests to the important role of NETs in cancer. NETs stimulate cancer-associated thrombosis, a symptom accompanying a very poor prognosis [26, 38]. The levels of circulating NETs were also measured in patients with hepatocellular carcinoma (HCC) by assessing the levels of the respective markers (DNA–histone complexes, double-stranded DNA, and NE). Markers of contact phase activation (factor XIIa and high-molecularweight kininogen) were measured in the same way. The levels of NETs and markers of contact phase activation were higher in patients with HCC compared to those in healthy subjects in [39]. Jung *et al*. revealed a correlation between the high levels of NET markers and hypercoagulation observed in patients with malignant pancreatic neoplasms [32]. Furthermore, the plasma levels of citrullinated histone H3 (H3-cit) were higher in late-stage cancer patients compared to those in healthy subjects while an elevated H3-cit level was found in the neutrophils of cancer patients. In addition, the plasma level of H3-cit in cancer patients did correlate with the levels of NETosis activators: NE, MPO, interleukins-6 and -8 [40, 41].

**An elevated level of NETs correlates with the presence of a tumor process**

Spontaneous intestinal neoplasia in mice correlates with the accumulation of immunosuppressive pro-oncogenic low-density neutrophils with an N2 phenotype, activation of the complement receptor C3a, and NET formation [42].

A positive correlation between an elevated plasma level of NETs and various tumor processes was revealed in studies that compared cancer patients and healthy subjects. Li *et al*. detected NETs in the lung tissue, peripheral blood, and sputum in patients with lung cancer [33]. In patients with colorectal cancer, the levels of NETs produced by neutrophils after *in vitro* stimulation were significantly higher than those in the control group consisting of healthy subjects and came with an unfavorable clinical outcome [10]. Park *et al*. demonstrated the presence of NETs in patients with breast cancer. NETs were also detected in lung metastases in this case; the highest percentage was revealed in patients with triple-negative breast cancer [11]. Identically, Tohme *et al*. [41] found that the amount of TANs and NETs in the histopathology specimens of hepatic metastases from colorectal cancer patients was increased compared to that in healthy subjects. Furthermore, high levels of citrullinated histones were also detected in tumors, being indicative of NETosis. The preoperative serum levels of MPO–DNA, a reliable marker of systemic NETosis [41], were higher in patients compared to those in healthy controls and were associated with a poor prognosis. Therefore, the serum levels of MPO–DNA can potentially be a prognostic marker in these patients [31].

**NETs and cancer cells adhere to each other**

**Fig. 2:**
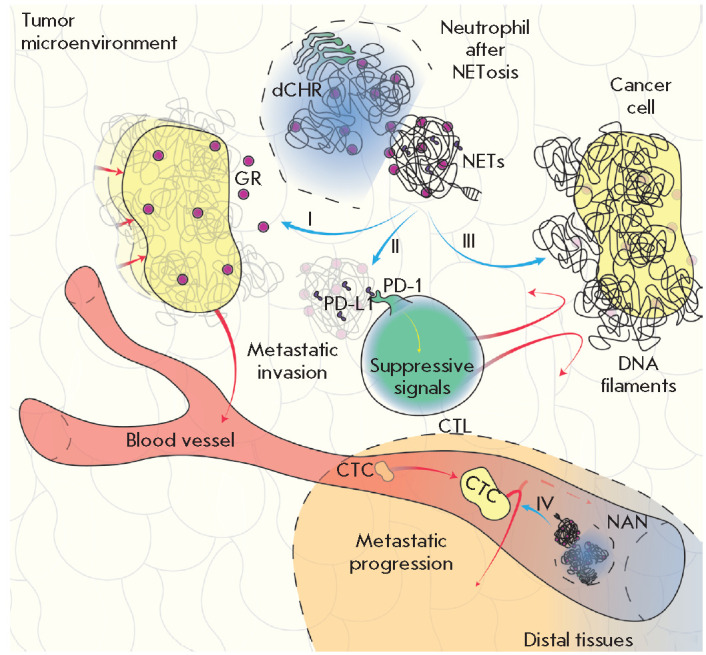
The diverse effects of NETs. NET granules contain fragments that promote dormant cancer cell awakening and change their phenotype to a metastatic one (I); NETs also contain suppressor molecules (PD-L1), which interact with cytotoxic cells and suppress their activity (II); DNA filaments, the key component of NETs, ensnare tumor cells, thus acting as a steric hindrance to the interaction with cytotoxic cells (III); the awakened cancer cells leave the microenvironment and enter blood vessels; these circulating cells are entrapped in distal tissues via NETs, which promotes metastasizing (IV). Abbreviations: dCHR – decondensed chromatin; NETs – neutrophil extracellular traps; GR – granule; PD-L1 – programmed death ligand 1; PD-1 – PD-L1 receptor; CTL – cytotoxic T lymphocyte; CTC – circulating tumor cell; NAN – neutrophil after NETosis

Along with exhibiting local tumor and systemic effects, NETs can promote metastasizing by entrapping circulating tumor cells (CTCs) (*Fig. 2, IV*) [43]. Adhesion of cancer cells to NETs and upregulated expression of integrin beta-1 both in cancer cells and in NETs, which seems to be a key factor of CTC adhesion to NETs, was demonstrated in mice with intraperitoneal sepsis mimicking postoperative inflammation. Treatment with DNase I inhibited this process [44]. In mouse models, NETosis and the entrapment of CTCs in lungs caused hepatic micrometastases [45]. Finally, NETs contributed to the development and progression of hepatic metastases after a surgical intervention [41]. Monti *et al*. [46] demonstrated that different cancer cell lines (HT1080, U-87MG, H1975, DU 145, PC-3, and A-431) can adhere *in vitro *to NETs formed from neutrophil- like cells through the integrins α_5_β_1_, α_v_β_3_ and α_v_β_5_ that were present on the cell surface. An excess of cyclic peptide RGD inhibited the adhesion of cancer cells to NETs to a level similar to that observed during hydrolysis of NETs by DNase I.

**NETs induce metastases**

In addition to all the functions described earlier, NETs awaken dormant cancer cells (*Fig. 2, I*). The involvement of NET in tumor recurrence was recently established [47]. Chronic lung inflammation caused by tobacco smoke or nasal instillation of a NETosis-activating lipopolysaccharide was found to promote the activation of dormant cancer cells and metastasizing. NETs were found bound to the extracellular matrix and triggered laminin cleavage and remodeling to give rise to a new surface epitope, which initiated the proliferation of dormant cells by activating integrin and transducing signals through the FAK/ERK/ MLCK/YAP kinase pathway. The *in vitro *and *in vivo* NET degradation by DNase I suppressed metastasizing. Monteiro *et al*. [47] assessed the ability of isolated NETs to change the phenotype of human breast cancer cells to a pro-metastatic one. NETs change the typical morphology of MCF7 cells from the epithelial phenotype to a mesenchymal one, when the migratory properties of a tumor are enhanced and there are typical signs of epithelial–mesenchymal transition (EMT) such as elevated levels of N-cadherin and fibronectin. Meanwhile, the E-cadherin level was found to decrease. Interestingly, NETs positively regulate the expression of genes encoding several factors associated with proinflammatory and pro-metastatic properties. Comparison of the Cancer Genome Atlas and RNA sequencing data revealed that specimens taken from patients with breast cancer show a significant correlation between the expression of the protumor genes and the expression of the genes whose products are involved in the interaction with neutrophils. Therefore, NETs drive the pro-metastatic phenotype in human breast cancer cells by activating the EMT program.

**NETs suppress the activity of cytotoxic cells**

In addition to the functions already listed above, an important function of NETs is that they “hide” cancer cells from cytotoxic immune ones. In their recent study, Melero *et al*. [48] showed that CXCL chemokines released by tumor cells induce NETosis in TANs. The resulting NETs envelop the tumor using DNA filaments to form a physical hindrance to any interaction between T cells or NK cells and tumors (*Fig. 2, III*). Furthermore, as established recently, NETs can contain suppressor molecules (e.g., PD-L1) and have a negative effect on the activity of cytotoxic lymphocytes (*Fig. 2, II*) [49]. A specific role in the study of NETs should be assigned to work on the treatment of cancer pathologies with the help of re-programmed T cells with induced cytolytic activity. CAR-T therapy of hematological cancer, taking into account the approaches of personalized medicine, is increasingly becoming a reality [50, 51]. At the same time, the possibilities of CAR-T therapy for solid tumors remain very limited [52]. It is likely that NETs, in this case, will become important in efforts to overcome the barriers to effective CAR-T therapy.

## METHODS FOR DETECTING AND INFLUENCING NETS

According to recent findings, NETs could turn into a promising therapeutic target for cancer. Judging by the crucial role played by NETs in enhancing the metastatic potential of malignant cells, patients prognosis can be improved by inhibiting NET formation and activity [11].

**Markers of NETs**

To perform clinical screening of NETs, the reference levels of NETosis need to be identified using a standardized procedure. However, a fully reliable method has not been reported in the literature yet. The simplest techniques for detecting NETs *in vivo *include measuring of the blood levels of NET-bound substances such as circulating cell-free DNA, H3-cit, NE, and MPO. Thus, the amount of circulating free DNA was measured in the serum specimens of patients with colorectal and breast cancer using simple nucleic acid staining [53, 54]. Although the amount of circulating DNA is known to correlate with the size and grade of breast tumor [55], the direct DNA staining technique was not specific enough in order to measure NETosis. The increased serum level of cell-free DNA (cfDNA) in cancer patients can also be related to other factors such as apoptotic and necrotic cells or the microorganisms passing into the systemic blood flow when permeability of the intestinal wall increases [56]. Hence, measuring circulating MPO–DNA conjugates is more specific to NET formation than for assessing the cfDNA level only [57]. H3-cit results from PAD4-mediated citrullination during NETosis and is the most specific marker of circulating NETs [58]. Furthermore, H3-cit can have prognostic significance, since Thalin *et al*. [40] have revealed that a high plasma level of H3-cit is a significant prognostic factor of short-term mortality in patients with late-stage cancer. Despite this, there were no significant differences in other NET-related markers, including NE and MPO, in severely ill patients with or without malignant neoplasms. The reason is that these enzymes can be released independently during neutrophil degranulation, in the absence of NET formation. These findings indicate that H3-cit currently remains the most reliable indicator of NETosis.

**NETs as a therapeutic target**

According to the review by Jorch and Kubes [59], the vast majority of experimental and clinical studies focusing on NETs were conducted for noncancer pathologies such as autoimmune or lung diseases, or the complications associated with autoimmune disorders. Autoimmune pathologies characterized by a high level of antibodies to DNA are of particular interest in terms of studying the role of NETs [60, 61, 62, 63, 64]. Studies involving patients with systemic lupus erythematosus (SLE) have shown that serum DNase I is important for the hydrolysis of NET chromatin. Moreover, in some patients with SLE, DNase I dysfunction causes severe renal damage, which reinforces the fact that the balance between NET formation and degradation is extremely important [65]. Based on these findings, DNase I was tested using experimental cancer models. Thus, treatment with DNase I mitigated disease severity in mouse models of breast cancer [36]. Furthermore, in the mouse model of intraperitoneal sepsis mimicking a postoperative inflammatory environment, DNase I disrupted* in vivo *interaction between NETs and circulating tumor cells [44]. Systemic administration of DNase I also reduced the number of metastases in the mouse model of metastatic lung cancer [45], while DNase I-coated nanoparticles exhibited an even stronger effect due to enzyme stabilization. The DNase I nanoparticles hydrolyzed NETs *in vitro *and inhibited the spread of metastatic breast cancer to the lungs *in vivo*, although it had no effect on the growth of the primary tumor [11, 66]. In a recent study [67], a novel method for increasing plasma activity of DNase I was demonstrated. DNase I gene transfer to hepatocytes mediated by adeno-associated viruses after a single intravenous injection in a mouse model of colorectal cancer suppressed metastases and increased the number of CD8^+^ T cells in the tumors [68, 69]. These encouraging results obtained using animal studies give grounds for performing clinical trials once DNase I can be used as an antitumor agent.

It would be reasonable to extend the application of the inhibitors of the molecules involved in NETosis and preventing NET formation currently employed for non-cancer pathologies so as to use these inhibitors on cancer patients after they have undergone clinical trials. These agents include NE inhibitors, which are used to treat the chronic obstructive pulmonary disease, and PAD4 inhibitors. These compounds can improve the clinical outcome for cancer patients [25] even though the commercially available PAD4 inhibitors (e.g., Cl^-^amidine) have a short half-life in blood serum [70]. Domingo-Gonzalez *et al*. proposed to use prostaglandin E2 (PGE2) as an alternative inhibitor of NET formation; through the prostaglandin receptors EP2 or EP4, prostaglandin negatively affects NETosis both in mice and in patients who have undergone hematopoieic stem cell transplantation [71]. Another study has shown either that PGE2 inhibits the NET formation induced both by cancer cells and PMA (probably due to the increased concentration of intracellular cAMP and reduced concentration of intracellular Ca^2+^ needed for NET formation) or that antithrombin significantly inhibits the NET formation induced by cancer cells [72]. Along with the NETosis inhibitors listed above, the NET inhibitor chloroquine was proved to reduce platelet aggregation, the level of circulating tissue factor (coagulation factor III), and hypercoagulation in mice with tumor. The same effects were uncovered in patients with cancer [37].

Unfortunately, clinical trials are far from being concluded, and the optimal method for affecting NETs is yet to be determined (NCT03781531, NCT04177576, NCT04294589, NCT01491230, and NCT01533779).

## CONCLUSIONS

The unique role played by NETs in carcinogenesis, including their ability to initiate neoplastic transformation, accelerate tumor growth and metastatic spread, not to mention enhance resistance to anticancer therapy, makes NETs a relevant therapeutic target. There is an increasing number of promising studies that focus on using various approaches to NETs degradation in oncology, including the use of DNase I. The application of DNase I implies that both NETs and cfDNA will undergo degradation, which is expected to ensure a more efficient inhibitory effect on cancer. The optimal approach to combatting NETs is yet to be identified; future research does need to focus on NETosis regulation and the balance between NET formation and degradation, so that NETs could be affected without disturbing the immune system functions. Furthermore, there is additional value in considering as cancer therapy disrupters tight junctions. They maintain the integrity of solid epithelial tumors and prevent the penetration of bulky agents, including T cells and NK cells, into the tumor’s depth. In the areas of the intercellular junction of epithelial cells protein desmoglein 2 is in action. It provides structural adhesion of neighboring cells [73]. Recombinant proteins called “junction openers” bind desmoglein 2. They cause a temporary and specific opening of tight junctions that allows various therapeutic agents to penetrate tumors [74, 75]. It seems possible that the combined use of DNase I and “junction openers” could increase the effectiveness of anticancer therapy, since it would facilitate the effective penetration of agents, including cytotoxic cells, into the depths of a malignant neoplasm.

## References

[1] Stepanov A.V., Belogurov A.A.J., Ponomarenko N.A., Stremovskiy O.A., Kozlov L.V., Bichucher A.M., Dmitriev S.E., Smirnov I.V., Shamborant O.G., Balabashin D.S. (2011). PLoS One..

[2] Ukrainskaya V.M., Rubtsov Y.P., Knorre V.D., Maschan M.A., Gabibov A.G., Stepanov A.V. (2019). Acta Naturae..

[3] Guryev E.L., Volodina N.O., Shilyagina N.Y., Gudkov S.V., Balalaeva I.V., Volovetskiy A.B., Lyubeshkin A.V., Sen’ A.V., Ermilov S.A., Vodeneev V.A. (2018). Proc. Natl. Acad. Sci. USA..

[4] Ukrainskaya V.M., Stepanov A.V., Glagoleva I.S., Knorre V.D., Belogurov A.A., Gabibov A.G. (2017). Acta Naturae..

[5] Sokolova E., Proshkina G., Kutova O., Shilova O., Ryabova A., Schulga A., Stremovskiy O., Zdobnova T., Balalaeva I., Deyev S. (2016). J. Control. Release..

[6] Brinkmann V., Reichard U., Goosmann C., Fauler B., Uhlemann Y., Weiss D.S., Weinrauch Y., Zychlinsky A. (2004). Science (80-.)..

[7] Branzk N., Lubojemska A., Hardison S.E., Wang Q., Gutierrez M.G., Brown G.D., Papayannopoulos V. (2014). Nat. Immunol..

[8] Berger-Achituv S., Brinkmann V., Abu-Abed U., Kühn L., Ben-Ezra J., Elhasid R., Zychlinsky A. (2013). Front. Immunol..

[9] Oklu R., Sheth R.A., Wong K.H.K., Jahromi A.H., Albadawi H. (2017). Cardiovasc. Diagn. Ther..

[10] Richardson J.J.R., Hendrickse C., Gao-Smith F., Thickett D.R. (2017). Int. J. Inflam..

[11] Park J., Wysocki R.W., Amoozgar Z., Maiorino L., Fein M.R., Jorns J., Schott A.F., Kinugasa-Katayama Y., Lee Y., Won N.H. (2016). Sci. Transl. Med..

[12] Delgado-Rizo V., Martínez-Guzmán M., Iñiguez-Gutierrez L., García-Orozco A., Alvarado-Navarro A., Fafutis-Morris M. (2017). Front. Immunol..

[13] Papayannopoulos V., Metzler K.D., Hakkim A., Zychlinsky A. (2010). J. Cell Biol..

[14] Wang Y., Li M., Stadler S., Correll S., Li P., Wang D., Hayama R., Leonelli L., Han H., Grigoryev S.A. (2009). J. Cell Biol..

[15] Yang H., Biermann M.H., Brauner J.M., Liu Y., Zhao Y., Herrmann M. (2016). Front. Immunol..

[16] Radic M., Neeli I. (2013). Front. Immunol..

[17] Hoppenbrouwers T., Autar A.S.A., Sultan A.R., Abraham T.E., van Cappellen W.A., Houtsmuller A.B., van Wamel W. J.B., van Beusekom H.M.M., van Neck J.W., de Maat M.P.M. (2017). PLoS One..

[18] Naffah de Souza C., Breda L.C.D., Khan M.A., Almeida S.R. de., Câmara N.O.S., Sweezey N., Palaniyar N. (2018). Front. Immunol..

[19] Douda D.N., Khan M.A., Grasemann H., Palaniyar N. (2015). Proc. Natl. Acad. Sci. USA..

[20] Lood C., Blanco L.P., Purmalek M.M., Carmona-Rivera C., De +Ravin S.S., Smith C.K., Malech H.L., Ledbetter J.A., Elkon K.B., Kaplan M.J. (2016). Nat. Med..

[21] Clark S.R., Ma A.C., Tavener S.A., McDonald B., Goodarzi Z., Kelly M.M., Patel K.D., Chakrabarti S., McAvoy E., Sinclair G.D. (2007). Nat. Med..

[22] Kenny E.F., Herzig A., Krüger R., Muth A., Mondal S., Thompson P.R., Brinkmann V., von Bernuth H., Zychlinsky A. (2017). Elife..

[23] Urban C.F., Ermert D., Schmid M., Abu-Abed U., Goosmann C., Nacken W., Brinkmann V., Jungblut P.R., Zychlinsky A. (2009). PLoS Pathog..

[24] Fadini G.P., Menegazzo L., Rigato M., Scattolini V., Poncina N., Bruttocao A., Ciciliot S., Mammano F., Ciubotaru C.D., Brocco E. (2016). Diabetes..

[25] Brinkmann V. (2018). J. Innate Immun..

[26] Cools-Lartigue J., Spicer J., Najmeh S., Ferri L. (2014). Cell. Mol. Life Sci..

[27] Sangaletti S., Tripodo C., Vitali C., Portararo P., Guarnotta C., Casalini P., Cappetti B., Miotti S., Pinciroli P., Fuligni F. (2014). Cancer Discov..

[28] Homa-Mlak I., Majdan A., Mlak R., Małecka-Massalska T. (2016). Postepy Hig. Med. Dosw. (Online)..

[29] Masucci M.T., Minopoli M., Carriero M.V. (2019). Front. Oncol..

[30] Powell D.R., Huttenlocher A. (2016). Trends Immunol..

[31] Yazdani H.O., Roy E., Comerci A.J., van der Windt D.J., Zhang H., Huang H., Loughran P., Shiva S., Geller D.A., Bartlett D.L. (2019). Cancer Research.

[32] Jung H.S., Gu J., Kim J.E., Nam Y., Song J.W., Kim H.K. (2019). PLoS One..

[33] Li Y., Yang Y., Gan T., Zhou J., Hu F., Hao N., Yuan B., Chen Y., Zhang M. (2019). Int. J. Oncol..

[34] Demers M., Krause D.S., Schatzberg D., Martinod K., Voorhees J.R., Fuchs T.A., Scadden D.T., Wagner D.D. (2012). Proc. Natl. Acad. Sci. USA..

[35] Demers M., Wagner D.D. (2013). Oncoimmunology..

[36] Cedervall J., Zhang Y., Huang H., Zhang L., Femel J., Dimberg A., Olsson A.K. (2015). Cancer Research..

[37] Boone B.A., Murthy P., Miller-Ocuin J., Doerfler W.R., Ellis J.T., Liang X., Ross M.A., Wallace C.T., Sperry J.L., Lotze M.T. (2018). BMC Cancer..

[38] Demers M., Wagner D.D. (2014). Semin. Thromb. Hemost..

[39] Seo J. Do., Gu J.-Y., Jung H.S., Kim Y.J., Kim H.K. (2019). Clin. Appl. Thromb..

[40] Thålin C., Lundström S., Seignez C., Daleskog M., Lundström A., Henriksson P., Helleday T., Phillipson M., Wallén H., Demers M. (2018). PLoS One..

[41] Tohme S., Yazdani H.O., Al-Khafaji A.B., Chidi A.P., Loughran P., Mowen K., Wang Y., Simmons R.L., Huang H., Tsung A. (2016). Cancer Research.

[42] Guglietta S., Chiavelli A., Zagato E., Krieg C., Gandini S., Ravenda P.S., Bazolli B., Lu B., Penna G., Rescigno M. (2016). Nat. Commun..

[43] Szczerba B.M., Castro-Giner F., Vetter M., Krol I., Gkountela S., Landin J., Scheidmann M.C., Donato C., Scherrer R., Singer J. (2019). Nature.

[44] Najmeh S., Cools-Lartigue J., Rayes R.F., Gowing S., Vourtzoumis P., Bourdeau F., Giannias B., Berube J., Rousseau S., Ferri L.E. (2017). Int. J. Cancer..

[45] Cools-Lartigue J., Spicer J., McDonald B., Gowing S., Chow S., Giannias B., Bourdeau F., Kubes P., Ferri L. (2013). J. Clin. Invest..

[46] Monti M., De Rosa V., Iommelli F., Carriero M.V., Terlizzi C., Camerlingo R., Belli S., Fonti R., Di Minno G., Del Vecchio S. (2018). Int. J. Mol. Sci..

[47] Martins-Cardoso K., Almeida V.H., Bagri K.M., Rossi M.I., Mermelstein C.S., König S., Monteiro R.Q. (2020). Cancers (Basel)..

[48] Teijeira Á., Garasa S., Gato M., Alfaro C., Migueliz I., Cirella A., de Andrea C., Ochoa M.C., Otano I., Etxeberria I. (2020). Immunity..

[49] Zhang H., van der Windt D.J., Ren J., Tsung A., Huang H. (2019). J. Immunol..

[50] Stepanov A.V., Markov O.V., Chernikov I.V., Gladkikh D.V., Zhang H., Jones T., Sen’kova A.V., Chernolovskaya E.L., Zenkova M.A., Kalinin R.S. (2018). Sci. Adv..

[51] Huang J., Alexey S., Li J., Jones T., Grande G., Douthit L., Xie J., Chen D., Wu X., Michael M. (2019). Leukemia..

[52] Kalinin R.S., Petukhov A.V., Knorre V.D., Maschan M.A., Stepanov A.V., Gabibov A.G. (2018). Acta Naturae..

[53] Agassi R., Czeiger D., Shaked G., Avriel A., Sheynin J., Lavrenkov K., Ariad S., Douvdevani A. (2015). Am. J. Clin. Pathol..

[54] Czeiger D., Shaked G., Eini H., Vered I., Belochitski O., Avriel A., Ariad S., Douvdevani A. (2011). Am. J. Clin. Pathol..

[55] Kohler C., Radpour R., Barekati Z., Asadollahi R., Bitzer J., Wight E., Bürki N., Diesch C., Holzgreve W., Zhong X.Y. (2009). Mol. Cancer..

[56] Bronkhorst A.J., Ungerer V., Diehl F., Anker P., Dor Y., Fleischhacker M., Gahan P.B., Hui L., Holdenrieder S., Thierry A.R. (2021). Hum. Genet..

[57] Yoo D., Floyd M., Winn M., Moskowitz S.M., Rada B. (2014). Immunol. Lett..

[58] Mauracher L.M., Posch F., Martinod K., Grilz E., Däullary T., Hell L., Brostjan C., Zielinski C., Ay C., Wagner D.D. (2018). J. Thromb. Haemost..

[59] Jorch S.K., Kubes P. (2017). Nat. Med..

[60] Gabibov A.G., Ponomarenko N.A., Tretyak E.B., Paltsev M.A., Suchkov S.V. (2006). Autoimmun. Rev..

[61] Belogurov A.J., Kozyr A., Ponomarenko N., Gabibov A. (2009). Bioessays..

[62] Durova O.M., Vorobiev I.I., Smirnov I.V., Reshetnyak A.V., Telegin G.B., Shamborant O.G., Orlova N.A., Genkin D.D., Bacon A., Ponomarenko N.A. (2009). Mol. Immunol..

[63] Gololobov G.V., Mikhalap S.V., Starov A.V., Kolesnikov A.F., Gabibov A.G. (1994). Appl. Biochem. Biotechnol..

[64] Kozyr A.V., Kolesnikov A.V., Zelenova N.A., Sashchenko L.P., Mikhalap S.V., Bulina M.E., Ignatova A.N., Favorov P.V., Gabibov A.G. (2000). Appl. Biochem. Biotechnol..

[65] Hakkim A., Fürnrohr B.G., Amann K., Laube B., Abed U.A., Brinkmann V., Herrmann M., Voll R.E., Zychlinsky A. (2010). Proc. Natl. Acad. Sci. USA..

[66] Hollmén M., Karaman S., Schwager S., Lisibach A., Christiansen A.J., Maksimow M., Varga Z., Jalkanen S., Detmar M. (2016). Oncoimmunology..

[67] Xia Y., He J., Zhang H., Wang H., Tetz G., Maguire C.A., Wang Y., Onuma A., Genkin D., Tetz V. (2020). Mol. Oncol..

[68] Alcazar-Leyva S., Ceron E., Masso F., Montano L.F., Gorocica P., Alvarado-Vasquez N. (2009). Med. Sci. Monit..

[69] Alekseeva L.A., Sen’kova A.V., Zenkova M.A., Mironova N.L. (2020). Mol. Ther. – Nucl. Acids..

[70] Knight J.S., Subramanian V., O’Dell A.A., Yalavarthi S., Zhao W., Smith C.K., Hodgin J.B., Thompson P.R., Kaplan M.J. (2015). Ann. Rheum. Dis..

[71] Domingo-Gonzalez R., Martínez-Colón G.J., Smith A.J., Smith C.K., Ballinger M.N., Xia M., Murray S., Kaplan M.J., Yanik G.A., Moore B.B. (2015). Am. J. Respir. Crit. Care Med..

[72] Shishikura K., Horiuchi T., Sakata N., Trinh D.A., Shirakawa R., Kimura T., Asada Y., Horiuchi H. (2016). Br. J. Pharmacol..

[73] Shilova O., Shilov E., Lieber A., Deyev S. (2018). J. Control. Release..

[74] Pitner R., Kim J., Davis-Bergthold J., Turner C., Stermann E., Adams J., Carter L., Ahlgren J., Fender P., Lieber A. (2019). Sci. Rep..

[75] Choi I.K., Strauss R., Richter M., Yun C.O., Lieber A. (2013). Front. Oncol..

